# Pathomechanisms of bone loss in rheumatoid arthritis

**DOI:** 10.3389/fmed.2022.962969

**Published:** 2022-08-17

**Authors:** Rajalingham Sakthiswary, Rajeswaran Uma Veshaaliini, Kok-Yong Chin, Srijit Das, Srinivasa Rao Sirasanagandla

**Affiliations:** ^1^Department of Medicine, Universiti Kebangsaan Malaysia Medical Centre, Kuala Lumpur, Malaysia; ^2^Department of Pharmacology, Universiti Kebangsaan Malaysia Medical Centre, Kuala Lumpur, Malaysia; ^3^Department of Human and Clinical Anatomy College of Medicine and Health Sciences Sultan Qaboos University, Muscat, Oman

**Keywords:** rheumatoid arthritis, bone, osteoporosis, cytokines, ligands, autoantibodies

## Abstract

Rheumatoid arthritis (RA) is an autoimmune disease, in which the inflammatory processes involve the skeletal system and there is marked destruction of the bones and the surrounding structures. In this review, we discuss the current concepts of osteoimmunology in RA, which represent the molecular crosstalk between the immune and skeletal systems, resulting in the disruption of bone remodeling. Bone loss in RA can be focal or generalized, leading to secondary osteoporosis. We have summarized the recent studies of bone loss in RA, which focused on the molecular aspects, such as cytokines, autoantibodies, receptor activator of nuclear kappa-β ligand (RANKL) and osteoprotegerin (OPG). Apart from the above molecules, the role of aryl hydrocarbon receptor (Ahr), which is a potential key mediator in this process through the generation of the Th17 cells, is discussed. Hence, this review highlights the key insights into molecular mechanisms of bone loss in RA.

## Introduction

### Bone remodeling mechanisms in RA

Physiological bone remodeling occurs through cell-mediated processes in response to biomechanical signals. The initial phase of bone resorption which is mediated by osteoclasts is followed by the recruitment of osteoblasts which mediate bone formation (1). Under normal circumstances, there is a tight coupling of osteoclastic and osteoblastic activities. Transforming growth factor-β (TGF-β) and bone morphogenetic proteins (BMPs) are bone growth factors which are sequestered into the bone matrix and have been strongly implicated in linking bone resorption and formation (2, 3). Besides, osteoclasts may independently modulate osteoblast differentiation and activity. There are other factors that influence physiologic bone remodeling include spingosine-1-phosphate, Wnt 10b and BMP6 that enhance bone formation (4) and semaphorin 4D that inhibits bone formation (5).

Rheumatoid arthritis (RA) is a form of chronic inflammatory arthritis with negative effects on skeletal remodeling. Periarticular bone erosions represent the radiographic hallmark of RA (6). Matrix metalloproteinases (MMPs) which are produced by synovial fibroblasts have been implicated in the degradation of extracellular matrix; mainly the proteoglycans and collagens of the articular cartilage (7). MMP-1 and MMP-3 levels were found to correlate strongly with joint erosions but there is a paucity of published data on the effects of MMPs on osteoclastogenesis (8–10). In active RA, disruptions in physiologic bone remodeling occur, whereby a mismatch in osteoclastic-osteoblastic activity results in enhanced bone resorption with a lack of compensatory bone formation. the processes that regulate the coupling of bone resorption and formation which usually occur under physiologic conditions. There is a mismatch in osteoclastic-osteoblastic activity resulting in enhanced bone resorption with a lack of compensatory bone formation. Beyond the disordered focal bone remodeling associated with the active synovitis of the affected joints, there is generalized axial and appendicular bone loss at distant sites which are not inflamed. Hence, there are 3 types of bone loss i.e., local, juxta-articular and systemic. While all these forms are related to inflammation, the underlying mechanisms for each type of bone loss vary. Systemic bone loss or osteoporosis in RA is caused by systemic inflammation, the use of glucocorticoid therapy and physical impairment while local bone loss in the form of joint erosions due to the effects of the synovial immune cells and cytokines (11). In RA, studies have consistently pointed out that biochemical markers of bone turnover have a significant positive correlation with the disease activity. The systemic bone loss is mediated by synovial cytokines with osteoclastogenic activity that enter the blood circulation and adversely affect the generalized bone remodeling (12).

**Figure 1:**
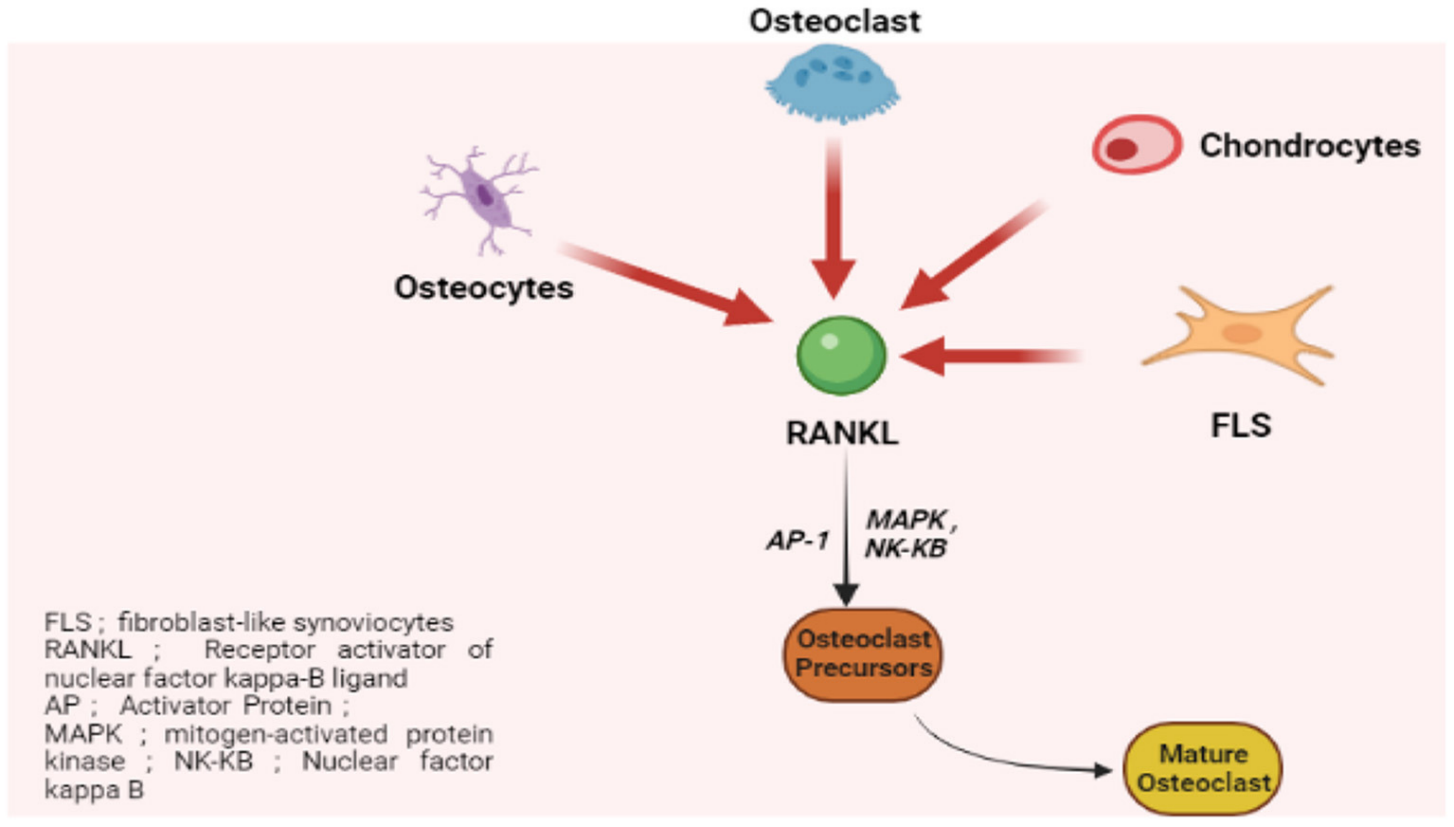
RANKL-dependent osteoclastogenesis.

The bone pannus interface in patients with RA exhibits a heterogeneous population of cells including osteoclasts. Animal models have implicated osteoclasts for the marginal joint erosions in RA. The rheumatoid synovium contains abundant osteoclast precursors and factors with potent pro-osteoclast differentiation and activation activity. Receptor activator of nuclear kappa-β ligand (RANKL), a member of the tumor necrosis factor ligand family, plays a vital role in osteoclastogenesis. RANKL is expressed by synovial fibroblasts and activated T cells in the joints of RA patients. Hence, treatment with osteoprotegerin (the soluble receptor that inhibits RANKL activity) results in marked inhibition of joint erosions (Figure 1).

In RA, an additional mechanism of bone adaptation involves the process of endochondral bone formation whereby there is replacement of cartilaginous matrix with mineralised bone. Local production of bone growth factors, including TGF-β and BMPs have been implicated in this process (13). Multiple cytokines, lipid mediators and growth factors have been shown to function as anabolic mediators of bone formation either *via* effects on osteoblasts and their precursors or *via* chondrogenesis. Of these mediators, particular attention has focused more recently on the wingless (WNT) signaling pathway (14). The Wnt ligands regulate bone formation *via* several distinct pathways, including the WNT/b-catenin pathway, the non-canonical WNT/planar cell polarity pathway and the WNT/calcium pathway. Signaling *via* the canonical pathway is initiated by the binding of WNT ligands to a dual receptor complex comprising low-density lipoprotein receptor-related protein (LRP) 5 or LRP6. This leads to the inactivation of a multiprotein β-catenin complex that targets β-catenin to proteosomal degradation. β-Catenin then accumulates in the cytoplasm and translocates to the nucleus, where it stimulates osteoblast differentiation. Activation of the canonical β-catenin pathway results in transcriptional activation of the osteoprotegerin (OPG) gene (15). OPG is a potent inhibitor of RANK ligand (RANKL), which is the key regulator of osteoclast differentiation and activation. The WNT signaling pathway is regulated by families of both activators and inhibitors. Sclerostin, is one of the most potent inhibitors of this pathway (16). The Dickkopf (DKK) proteins and sclerostin, as well as soluble frizzled related protein play a role in modulating the pattern of bone repair and regeneration in RA (17).

## Receptor activator of nuclear kappa-β ligand (RANKL)/osteoprotegerin (OPG)

**Figure 2:**
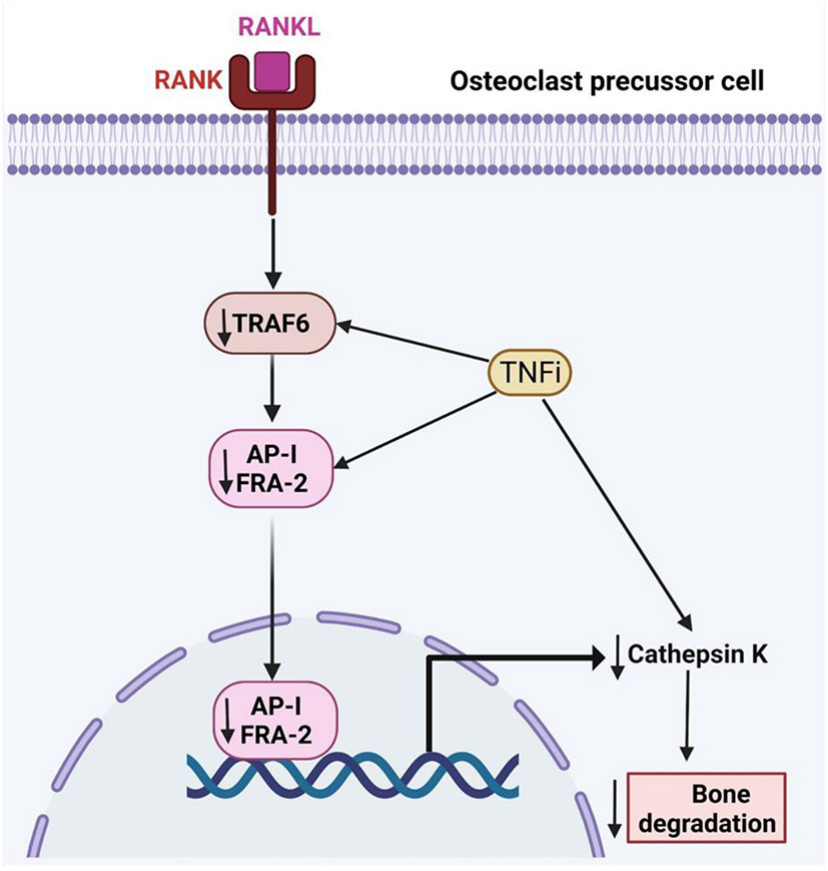
The effects of tumor necrosis factor inhibitors on RA-related bone loss. TRAF6, TNF-receptor associated factor-6; FRA-2, Fos-related antigen-2; AP-1, Activator protein 1; RANK, receptor activator of nuclear factor kappa-B; RANKL, Receptor activator of nuclear factor kappa-B ligand.

The bone remodeling process is delicately orchestrated by osteoclasts and osteoblasts through multiple signaling pathways. The RANKL/OPG is the most recognized axis in mediating the formation of osteoclasts (18). Osteoblasts secrete RANKL that binds with RANK on the osteoclasts precursors to promote their differentiation into mature osteoclasts with bone-resorption ability. Apart from osteoblasts, hypertrophic chondrocytes and osteocytes are the major sources of RANKL during growth and maturation (19). In patients with RA and animals with experimentally induced inflammatory arthritis, fibroblast-like synoviocytes (FLS) represent a major source of RANKL (20, 21). Briefly, the binding of RANKL to RANK recruits adaptor protein tumor necrosis factor receptor and activates mitogen-activated protein kinases, nuclear factor-kappa β and activator protein-1. Coupled with immunoreceptor tyrosine-based activation motif-mediated calcium ion signaling, this eventually leads to activation and amplification nuclear factor of activated T-cells cytoplasmic 1, which transcribes genes that stimulate osteoclast differentiation and bone resorption (22, 23). OPG acts as a decoy receptor to bind with RANKL, thus preventing the activation of RANK-RANKL signaling (23, 24) (Figure 2).

The expression of RANKL and OPG is triggered by various factors, including inflammatory cytokines such as tumor necrosis factor (TNF)-α, interleukin (IL)-1, IL-6, IL-17 present in high levels among patients with RA (25). Of note, IL-6 and soluble IL-6 receptor induce RANKL expression in FLS and they are vital for the TNF-α and IL-17 mediated RANKL expression (26). A recent study revealed that the level of lymphotoxin-α, a close homolog of TNF-α, is higher in patients with RA compared to those with OA. It can induce RANKL expression in chondrocytes in a manner like TNF-α, which subsequently promotes the differentiation of peripheral mononuclear cells into osteoclast-like cells *in vitro* (27). Thus, these factors could contribute to increased RANKL levels leading to bone erosions; observed in RA patients. In tissues samples of patients with RA, RANKL and OPG are co-expressed at the sites of articular bone erosion, with RANK-positive multinucleated cells (28). In addition, RANKL expression by FLS is shown to play a more significant role in RA-associated bone erosion based on a study using cell-specific RANKL knockout animals (29). Conventional anti-RA agents like methotrexate have been shown to prevent bone resorption by reducing serum soluble RANKL levels (30).

Epidemiological studies have reported that RANKL level is higher in patients with RA compared to normal controls (31), and correlates with Larsen score and radiological progression (31). A meta-analysis showed that RANKL gene rs2277438 polymorphism increases RA risk, while RANK gene rs1805034, OPG gene rs3102735, rs2073618, rs3134069 polymorphism were not related to RA risk (32). A Polish study revealed that RANK rs8086340 single nucleotide polymorphism affects disease susceptibility while RANK rs8086340 and RANKL rs7325635 GG were associated with lower C-reactive protein levels. RANKL rs8086340 and rs1805034 CC homozygotes were associated with higher alkaline phosphatase levels compared to other phenotypes whereas RANKL rs7988338 GG homozygotes were associated with a lower number of swollen joints compared to the A allele (33). A meta-analysis of three French cohorts found that the OPG gene G allele of rs2073618 is associated with bone erosion defined by the Sharp score, but not RANKL rs7325635 gene allele (34).

Given the important role of RANKL in RA, denosumab, an antibody against RANKL, could ameliorate the effects of RANKL on bone damage. Individual randomized controlled trials have demonstrated the efficacy of denosumab in reducing new bone erosion and promoting bone erosion healing in patients with RA (35, 36). Discontinuation of denosumab among patients with RA receiving glucocorticoids leads to a gradually increased bone remodeling and decreased bone mineral density (BMD) to baseline level after 12 months (37). These data show that inhibition of RANKL activity is a promising approach to reduce bone damage in patients with RA. A study on Fc-osteoprotegerin infusion in animals with collagen-induced arthritis showed that the treatment can prevent bone erosion by inhibiting osteoclast formation, but not cartilage erosion (38). Table 1 summarizes the recent studies on RANKL/OPG in RA.

**Table 1:** Recent evidence on the association between RANKL/OPG and RA.

**Study**	**Study design**	**Key findings**
Komatsu et al. (21)	Animal study	RANKL from bone marrow plasma cells contributed to periarticular bone loss RANKL from synovial fibroblasts contribute to joint erosion
Perpetuo et al. (44)	Interventional study Subjects: Patients with RA and DAS28 >3.2 Treatment: Methotrexate for at least 6 months.	↓ RANK and monocyte activation markers with methotrexate ± low-dose prednisolone treatment. ↓ bone resorption from osteoclast-like cells differentiated from peripheral blood mononuclear cells *in vitro* after treatment. ↓ serum RANKL level after treatment. Serum OPG level was not changed.
Boman et al. (31)	Longitudinal study Subjects: Patients with early RA and were symptomatic <1 year. Followed up for 24 months	↑ RANKL in patients compared to controls. ↑ RANKL in anti-CCP-positive compared to seronegative patients RANKL level correlated positively with Larsen score at baseline & 24 months RANKL level correlated positively with radiological progression at 24 months
Yang et al. (32)	Case-control study Case: 574 patients with RA: 804 controls.	RANK gene rs1805034 was not related to risk of developing RA.
	Meta-analysis 9 studies involving Asians and Caucasians	RANKL gene rs2277438: ↑ RA risk. RANK gene rs1805034: not related to RA risk. OPG gene rs3102735, rs2073618, rs3134069: not related to RA.
Wielińska et al. (33)	Longitudinal studies Patients with RA, DAS28 > 5.1, qualified for TNFα inhibitor therapy Followed up for 12 weeks	↑ RANK rs8086340-G allele in patients than controls. ↓ CRP levels in RANK rs8086340 and RANKL rs7325635 GG homozygotes vs. other phenotypes. ↑ alkaline phosphatase level in RANKL rs8086340 and rs1805034 CC allele homozygotes vs. other phenotypes. ↓ number of swollen joints in RANKL rs7988338 GG homozygotes compared to A allele.
Ruyssen-Witrand et al. (34)	Meta-analysis Three Frech cohorts: Etude de Suivi des PolyArthrites Indifférenciées Récentes (ESPOIR) (*n* = 632) Rangueil Midi-Pyrénées (RMP) (*n* = 249) French Rheumatoid Arthritis Genetic Consortium (FRAGC) (*n* = 590)	↑ bone erosion in OPG rs2073618 G allele
Hu et al. (89)	Meta-analysis 10 studies with 1,758 patients	↓ modified total Sharp score and erosion score in denosumab-treated group.
So et al. (35)	Randomized controlled trial Subjects: Patients with RA and DAS28 ≤ 5.1. Treatment: subcutaneous denosumab 60 mg or placebo once every 6 months for 24 months	↑ new erosion and erosion progression in the placebo group after 24 months. ↑ erosion healing in the denosumab group at 24 months. No significant changes in joint space parameters, van der Heijde-Sharp erosion score, DAS28 and HAQ-DI between the two groups.
Takeuchi et al. (36)	Randomized controlled trial Subjects: Patients with RA receiving conventional synthetic disease-modifying antirheumatic drugs. Treatment: denosumab 60 mg every 3 months, denosumab every 6 months or placebo for 12 months.	↓ modified total Sharp score in denosumab-treated group. ↓ bone erosion score in denosumab-treated group. No difference in joint space narrowing score.
Saag et al. (37)	Randomized controlled trial Patients: Patients with RA Treatment: Denosumab 60 or 180 mg or placebo, every 6 months for 12 months. Subjects were followed up for an additional 12 months after discontinuation	↓ CTX and PINP in both denosumab groups (vs baseline). ↑ CTX following denosumab discontinuation, but not significantly different from level during treatment.

*RANKL, Receptor activator of nuclear factor kappa-β ligand; CTX 1, carboxy-terminal crosslinked telopeptide of type 1 collagen; CRP, C-reactive protein; CCP, citrullinated cyclic peptide; ↓, reduced; ↑, increased; DAS 28, 28-joints based disease activity score*.

## The central role of cytokines

The RANKL/OPG ratio determines the physiological balance of bone formation and resorption, with a higher ratio favoring increased bone resorption. A higher RANKL/OPG ratio is associated with increased radiographic damage in RA patients (39). Various proinflammatory cytokines regulate the expression of RANKL and OPG including TNFα, IL-6 and IL-1 (40). Table 2 summarizes the findings of recent studies on cytokine related bone loss in RA.

**Table 2:** Recent studies on cytokines-related bone loss in RA.

**Article**	**Study design**	**Key findings**
Jura-Poltorak et al. (43)	Cross sectional 50 female RA patients	In a 15-months anti-inflammatory treatment with TNFα blockers, increased bone formation markers i.e., C- and N-terminal propeptides of type I procollagen (PICP, PINP) and reduced bone resorption markers i.e., C- and N-terminal cross-linking telopeptides of type I collagen (CTX-I, NTX-I) were observed.
Perpetuo et al. (44)	Cross sectional 17 patients with RA, evaluated before and after starting TNF inhibitors therapy, were included in this study.	With TNFα inhibitors therapy, patients had reduced RANKL surface expression in B-lymphocytes and the frequency of circulating classical CD14brightCD16– monocytes. Apart from serum levels of RANKL, RANKL/OPG ratio, CTX-I, TRAF6 and cathepsin K showed reduction with TNFα inhibitors.
Matsuura et al. (49)	*In vivo* animal study Lipopolysaccharide (LPS) was injected into the calvarial periosteum of mice to induce inflammatory bone destruction. The sample size was not available	Anti-IL-6 receptor antibody and anti-TNFα antibody therapy affected mature osteoclasts and switched bone-resorbing osteoclasts to non-resorbing cells.
O'Brien et al. (50)	Longitudinal 24 mice were treated with either RANKL, or TNFα plus IL-6. Osteoprotegerin, anti-IL-6 receptor antibody and hydroxyurea were used to block RANKL, the IL6 receptor and cell proliferation, respectively.	Reduction of bone erosion and osteoclast formation in arthritic mice with inducible deficiency of RANK. TNFα,IL-6, but not RANKL, induced osteoclast formation in bone marrow and synovial cultures from RANK-deficient animals.
Polzer et al. (90)	*In vitro* studies Human TNFα mice (hTNFtg) were crossed with mice lacking IL-1. 32 animals were included in clinical, histological and cellular analyses and animals were killed at 12 weeks of age by cervical dislocation.	Lack of IL-1 completely reversed increased osteoclast formation and bone resorption in hTNFtg mice and the increased levels of RANKL in these mice. These data shows that IL-1 is essential for TNF-mediated bone loss. Despite TNF-mediated inflammatory arthritis, systemic bone is fully protected by the absence of IL-1.
Saidenberg-Kermanac'h et al. (25)	*In vivo* Study DBA/1 mice (*n* = 28) were immunized with bovine type II collagen to induce arthritis and subsequently treated with OPG-Fc or anti-TNFα antibody or both.	Systemic OPG and anti-TNFα antibody therapy prevented bone loss in arthritic mice through distinct mechanisms involving decreased bone resorption and preserved bone formation.
Gulyás et al. (91)	Longitudinal Study 36 RA and 17 Ankylosing Spondylitis patients undergoing 1-year etanercept or certolizumab-pegol therapy were studied.	Anti-TNFα antibody therapy halted further bone loss over 1 year. In general, anti-TNF antibody therapy significantly increased P1NP, SOST levels, and the P1NP/βCTX ratios, while decreased DKK-1 and CathK production at different time points in most patient subsets.
Zwerina et al. (92)	*In vivo* Study A total of 64 mice were examined in this study. Human TNF-transgenic (hTNFtg) mice were treated with anti-TNF antibody (infliximab), IL-1 receptor antagonist (IL-1Ra; anakinra), or OPG either alone or in combinations of 2 agents or all 3 agents.	Bone erosion was effectively blocked by anti-TNF antibody (-79%) and OPG (-60%), but not by IL-1 receptor antagonist monotherapy. The combination of anti-TNF with IL-1 receptor antagonist however, completely blocked bone erosion (-98%). Inhibition of bone erosion was accompanied by a reduction of osteoclast numbers in the synovial tissue.
Binder et al. (93)	*In vivo* and *in vitro* Study The effects of TNF inhibition on osteoclast precursors as well as local bone destruction *in vivo* were assessed by treating TNF-transgenic mice with different doses of adalimumab. Sample size not available.	TNF stimulated osteoclastogenesis mainly by increasing the number of osteoclast precursor cells *in vitro*. In the hTNF-transgenic mouse model of destructive arthritis, low-dose TNF-inhibiting therapy with adalimumab had no effect on synovial inflammation but significantly inhibited local bone destruction and the generation of osteoclasts.
Axmann et al. (94)	*In vivo* and *in vitro* Study The efficacy of a murine antibody against IL-6 in blocking osteoclast differentiation of mononuclear cells stimulated with RANKL was tested. In addition, arthritic human TNFalpha-transgenic mice were treated with anti-IL-6 antibody, and osteoclast formation and bone erosion were assessed in arthritic paws. Each group consists of 8 animals.	Blockade of IL-6 dose dependently reduced osteoclast differentiation and bone resorption in monocyte cultures stimulated with RANKL or RANKL plus TNF. In human TNF -transgenic mice, IL-6 blockade did not inhibit joint inflammation, but it strongly reduced osteoclast formation in inflamed joints as well as bone erosions.
Lange et al. (95)	Open-label prospective study 26 patients with persistently active RA were treated with infliximab.	After 12 months of infliximab therapy, there was a significant increase in BMD in the spine and the femoral neck. There was a significant increase in osteocalcin serum levels between baseline and after 12 months (*P* < 0.01) and a significant decrease in the marker for bone resorption (*P* < 0.01) but no change in serum calcium was observed.

*RANKL, Receptor activator of nuclear factor kappa-B ligand; BMD, bone mineral density; TNF, tumor necrosis factor; CTX 1, carboxy-terminal crosslinked telopeptide of type 1 collagen; P1NP, procollagen type I N-propeptide (PINP); SOST, sclerostin; DKK-1, Dickkopf WNT signaling pathway inhibitor 1: CathK, cathepsin K*.

### TNFα

TNFα promotes osteoclastogenesis by promoting the expression of the essential osteoclast differentiation factor, RANKL, and/or OPG, by bone marrow stromal cells (41, 42). Jura-Półtorak et al. demonstrated that the 15-month anti-inflammatory treatment with TNFα inhibitors was associated with increased bone formation markers i.e., C- and N-terminal propeptides of type I procollagen (PICP, PINP) and reduced bone resorption markers i.e., C- and N-terminal cross-linking telopeptides of type I collagen (CTX-I, NTX-I) (43). In keeping with these findings, Perpetuo et al. disclosed that after TNFα inhibitor therapy, patients had reduced RANKL surface expression in B-lymphocytes and the frequency of circulating classical CD14^bright^CD16^−^ monocytes. Apart from the serum levels of RANKL, RANKL/OPG ratio, CTX-I, TRAF6 and cathepsin K showed a reduction with TNFα inhibitors (44).

### IL-6

IL-6 has a wide variety of biological effects in RA such as induction of proliferation of B cells and plasma cells, enhancement of production of acute-phase protein by hepatocytes and stimulation of differentiation of T helper cells. IL-6 is one of the culprit cytokines responsible for joint destruction in RA.

In murine bone marrow cells, IL-6 suppresses osteoclast differentiation through inhibition of the nuclear factor of activated T cells cytoplasmic 1 (NFATc1) (45). Besides, animal studies have suggested that IL-6 is essential for the differentiation from naïve T cells to Th17 cells (46, 47). Th17 cells, a subset of T-helper cells, are also known to express RANKL (48). Following treatment with anti-IL-6 receptor antibodies in murine models of arthritis, osteoclasts tend to transform into non-resorbing cells (49). Recent studies have also reported that co-stimulation of IL-6 and TNFα may trigger osteoclast differentiation from bone marrow-derived macrophages. This form of osteoclastogenesis is considered to occur in a RANKL-independent manner, given that it is not inhibited by OPG (50).

### IL-1

The IL-1 family of ligands includes 11 members, with IL-1β as the main culprit in many inflammatory conditions. The inactive IL-1β precursor is cleaved by caspase-1 into an active cytokine (51), which binds to type I (IL-1RI) and type II (IL-1RII) specific receptors. IL-1β is a strong stimulator of osteoclastogenesis (52) through upregulation of the production of RANKL and downregulation of OPG (53). Besides, IL-1β increases prostaglandin synthesis (54), which is a potent resorption stimulus (10). Prostaglandins, such as prostaglandin E2 (PGE2), may mediate the upregulation of RANKL by activating cell-surface receptors (55). Furthermore, IL-1β also stimulates osteoclast activity by increasing the production of macrophage colony-stimulating factor (M-CSF) and inhibiting osteoclast apoptosis. IL-1β has an intimate relationship with TNF-α and experimental evidence suggests that blocking IL1β and TNF-α may completely halt bone resorption (56).

**Figure 3:**
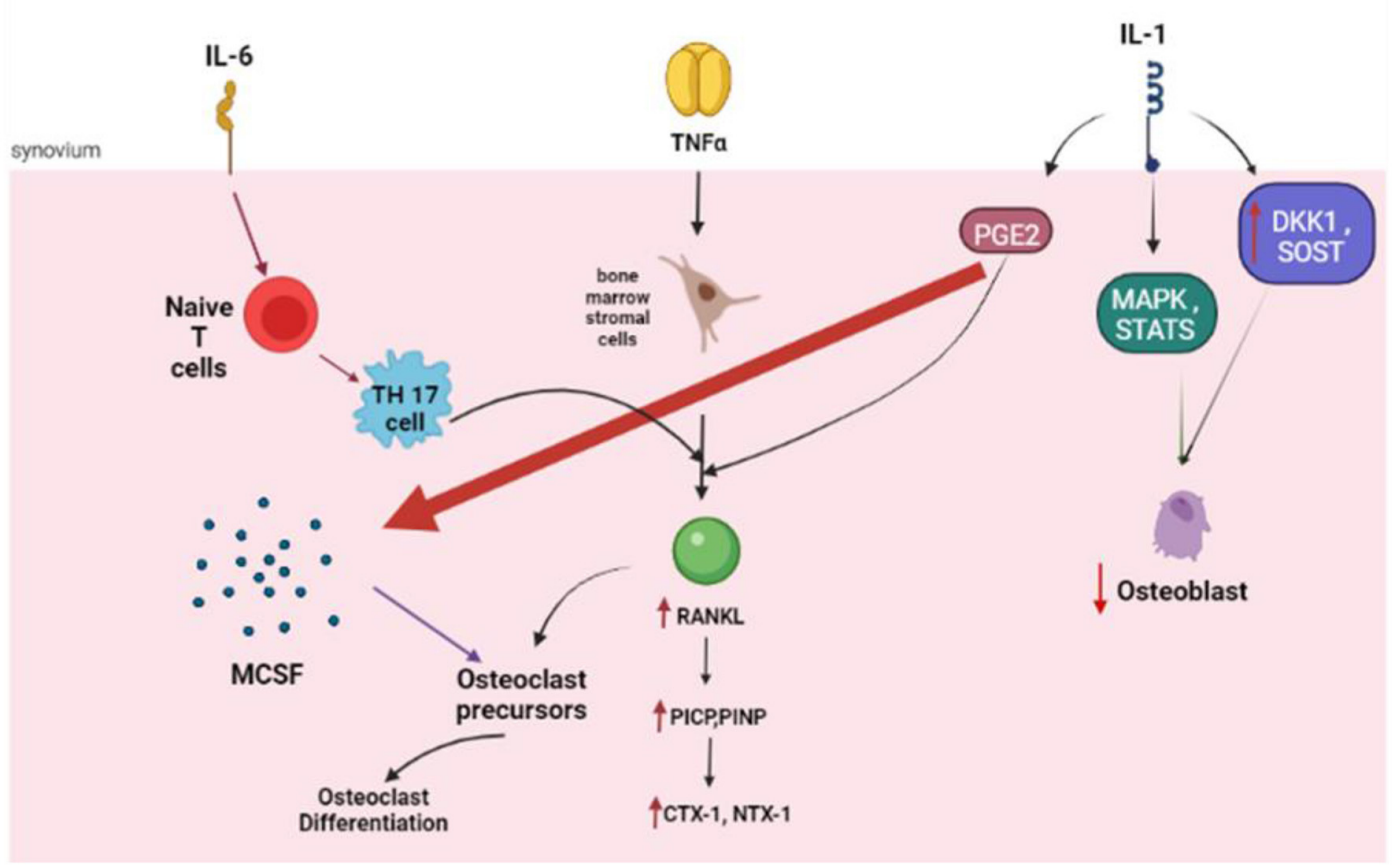
The role of the key cytokines in osteoclastogenesis. IL, Interleukin; NF, Tumor Necrosis factor; RANKL, Receptor activator of nuclear factor kappa-B ligand; PICP, propeptides of type I procollagen; PINP, N-terminal propeptides of type I procollagen; CTX 1, carboxy-terminal crosslinked telopeptide of type 1 collagen; NTX 1, amino-terminal crosslinked telopeptide of type 1 collagen; PGE, prostaglandin; MAPK, mitogen-activated protein kinase; DKK1, Dickkopf WNT signaling pathway inhibitor 1; SOST, Sclerostin.

Apart from enhancing osteoclastogenesis, IL-1β strongly inhibits osteoblastogenesis. The inhibition of osteoblast activity is modulated *via* mitogen-activated protein kinase (MAPK), by activated signal transducers and activators of transcription (STATs). IL-1β also upregulates DKK1- and sclerostin, which may inhibit the production of osteoblasts (57). In a Chinese population of patients with postmenopausal osteoporosis, there was a strong association between the Taq I IL-1β exon 5 gene polymorphism and a reduced BMD (58) (Figure 3).

### Other cytokines

Many other cytokines such as IL-22, IL-9, and IL-17A have been implicated to a lesser extent in RA-related bone loss (59–61). IL-17A which has a well-established role in the pathogenesis of enthesitis in spondyloarthritides. It exerts its pathogenic effects on FLS *via* IL-17/IL-17RA/STAT-3 signaling. IL-17 was found to have osteoclastogenic potential through the upregulation of RANKL (59). In a similar manner, IL-9 stimulation was reported to significantly enhance M-CSF -RANKL-mediated osteoclast formation and differentiation. IL-9 regulates the expression of genes in the metabolic pathways and the expression of matrix metalloproteinases (MMPs), which are responsible for bone degradation (60).

## Autoantibodies and bone resorption

Autoantibodies, namely rheumatoid factor (RF) and anti-citrullinated cyclic peptide (anti-CCP), play pivotal roles in RA for diagnostic and prognostic purposes. Seropositive disease is associated with a more severe and aggressive disease course (62). Emerging evidence suggests an association between anti-CCP and bone resorption. Clinical studies have consistently revealed that anti-CCP positivity was related to an increased risk of major fracture, lower BMD and osteopenia (63, 64). Bugatti et al. reported that anti-CCP positivity negatively affected the Z-scores of the spine and hip. The above association was observed even at low levels of RF [adjusted OR (95 % CI) 2.65 (1.01 to 7.24)], but was further increased by concomitant high RF [adjusted OR (95 % CI) 3.38 (1.11 to 10.34)] (64). This finding lends credence to the notion that autoantibodies may have a direct causative role in bone remodeling. In a Spanish cohort of patients with early arthritis and a median disease duration of 5.1 months, anti-CCP positivity remained significantly associated with lower BMD at the lumbar spine, femoral neck, and hip but not at the metacarpophalangeal joints despite adjustment for gender, age and body mass index (65). Taken together, these results may suggest that autoantibodies tend to induce systemic bone loss earlier than local or articular bone loss. Table 3 summarizes the findings of recent studies on anti-CCP and bone loss. However, there is a lack of data on the relationship between RF and bone loss.

**Table 3:** Recent studies on the association between anti-CCP and bone mineral density.

**Article**	**Study design**	**Key findings**
Cheng et al. (96)	Cross sectional The participants were categorized into two groups according to anti-CCP-positive (anti-CCP+) or anti-CCP– status and into four groups (I–IV) according to the anti-CCP level quartiles.	Compared with anti-CCP– patients, anti-CCP+ patients had a significantly higher 10-year probability of major fracture and a significantly lower BMD of the femoral neck (*p* = 0.0196). The rates of osteoporosis and previous fracture were comparable. The BMD and 10-year probability of major fracture among the groups were significantly different.
Hafstrom et al. (97)	Retrospective Baseline data from the BARFOT (Better Anti-Rheumatic PharmacOTherapy) cohort, which consists of patients with RA with a disease duration of 1 year or less.	Patients positive for anti-CCP had significantly more frequent osteopenia in the femoral neck and Ward's triangle compared with anti-CCP-negative patients (*p* = 0.016 and 0.003, respectively). This difference was found in men at any anti-CCP titer, but in women, osteopenia in these hip locations was found only in those with high anti-CCP titers (> 500 IU/ml). Anti-CCP was not associated with osteopenia in the lumbar spine or the metacarpal bones. In multiple logistic regression analyses, anti-CCP was independently associated with osteopenia in the femoral neck and/or Ward's triangle.
Wysham et al. (98)	Cross sectional Demographic, clinical, laboratory and functional variables were collected at study visits.	Age and high anti-CCP positivity were negatively associated with BMD after controlling for other variables (β = −0.003 and −0.055, respectively, *p* < 0.05). In highly-positive anti-CCP participants, increasing anti-CCP levels were associated with a negative linear trend in BMD (β = −0.011, *p* = 0.026).
Kurowska et al. (63)	Cross sectional. Bone marrow samples taken from the femur during a hip replacement surgery performed as part of normal clinical care. Paired peripheral blood samples were collected 1–1.5 h before the hip joint prosthesis implantation.	Anti-CCP present in RA bone marrow was associated with increased amounts of TRAP5b, cathepsin K and CTX-I in this location. Levels of IL-8, the key mediator of anti-CCP-induced bone resorption, were also elevated in bone marrow containing anti-CCP antibodies and positively correlated with TRAP5b and cathepsin K concentrations. Higher levels of TRAP5b, cathepsin K, CTX-I and IL-8 in bone marrow compared to peripheral blood indicate local generation of these molecules.
Amkreutz et al. (99)	Longitudinal Dual x-ray absorptiometry of the lumbar spine and left hip was performed in 408 Dutch patients with early RA during 5 years of follow-up and in 198 Swedish patients with early RA during 10 years of follow-up.	In the Dutch cohort, significantly lower BMD at baseline was observed in anti CCP-positive patients compared to anti CCP-negative patients. In the Swedish cohort, anti-CCP-positive patients tended to have a higher prevalence of osteopenia at baseline (*P* = 0.04).
Ahmad et al. (100)	Cross sectional Eligible patients had known BMD, as measured by digital X-ray radiogrammetry (DXR-BMD), and anti-CCP2 antibody measurements at the same time point or within 6 months.	DXR-BMD was lower in the anti-CCP2 + ve vs. the anti-CCP2-ve groups. DXR-BMD decreased with increasing anti-CCP2 titer (*P* < 0.001 for left and right hands).
Bugatti et al. (64)	Cross sectional Systemic bone mineral density (BMD) was measured in the lumbar spine and the hip in 155 consecutive treatment-naïve patients with early RA (median symptom duration 13 weeks).	The anti-CCP positivity negatively affected the Z-scores of the spine and hip. The above association was observed even at low levels of RF [adjusted OR (95 % CI) 2.65 (1.01 to 7.24)], but was further increased by concomitant high RF [adjusted OR (95 % CI) 3.38 (1.11 to 10.34)].
Llorente et al. (65)	Cross sectional BMD was measured using dual X-ray absorptiometry. Anti-CCP titers were determined through enzyme immunoassay	Anti-CCP positivity remained significantly associated with lower bone density at the lumbar spine, femoral neck, and hip but not at the metacarpophalangeal joints despite adjustment for gender, age and body mass index

*BMD, bone mineral density; CCP, citrulinated cyclic peptide; TRAP5b, Tartrate-resistant acid phosphatase 5b; CTX-I, carboxy-terminal crosslinked telopeptide of type 1 collagen; RF, Rheumatoid Factor*.

The levels of the active TRAP5b isoform, which represent the number and activity of osteoclasts (35, 36), and cathepsin K, which degrades the bone matrix proteins (37, 38), were both found to be significantly higher in the bone marrow samples with anti-CCP. The anti-CCP-positive bone marrow samples also contained increased concentrations of CTX-I, which is produced as a result of the enzymatic activity of cathepsin K (66, 67).

The key orchestrator of anti-CCP-dependent osteoclastogenesis is IL-8 (68). The concentration of this cytokine increases during osteoclast differentiation and in the presence of anti-CCP. Bone marrow samples from RA patients revealed increased IL-8 levels in anti-CCP positive bone marrow. TRAP5b and cathepsin K levels were found to correlate positively with IL-8 concentration (63).

**Figure 4:**
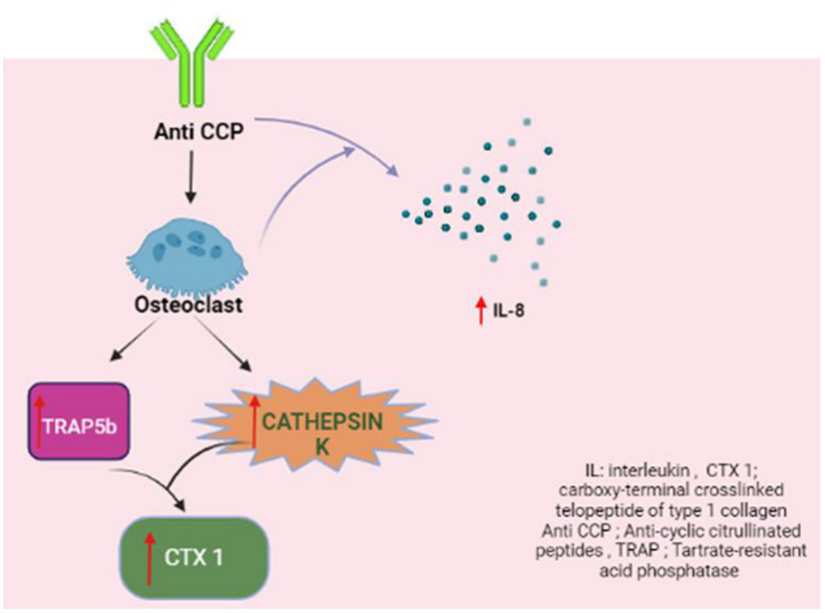
Anti-CCP and osteoclastogenesis.

Apart from RF and anti-CCP, anti-carbamylated protein antibodies, which are not routinely tested in clinical practice, were found to have an independent association with lower BMD in the spine and hip but not the metacarpophalangeal joint (69) (Figure 4).

## Role of aryl hydrocarbon receptor in RA-associated bone erosion

**Figure 5:**
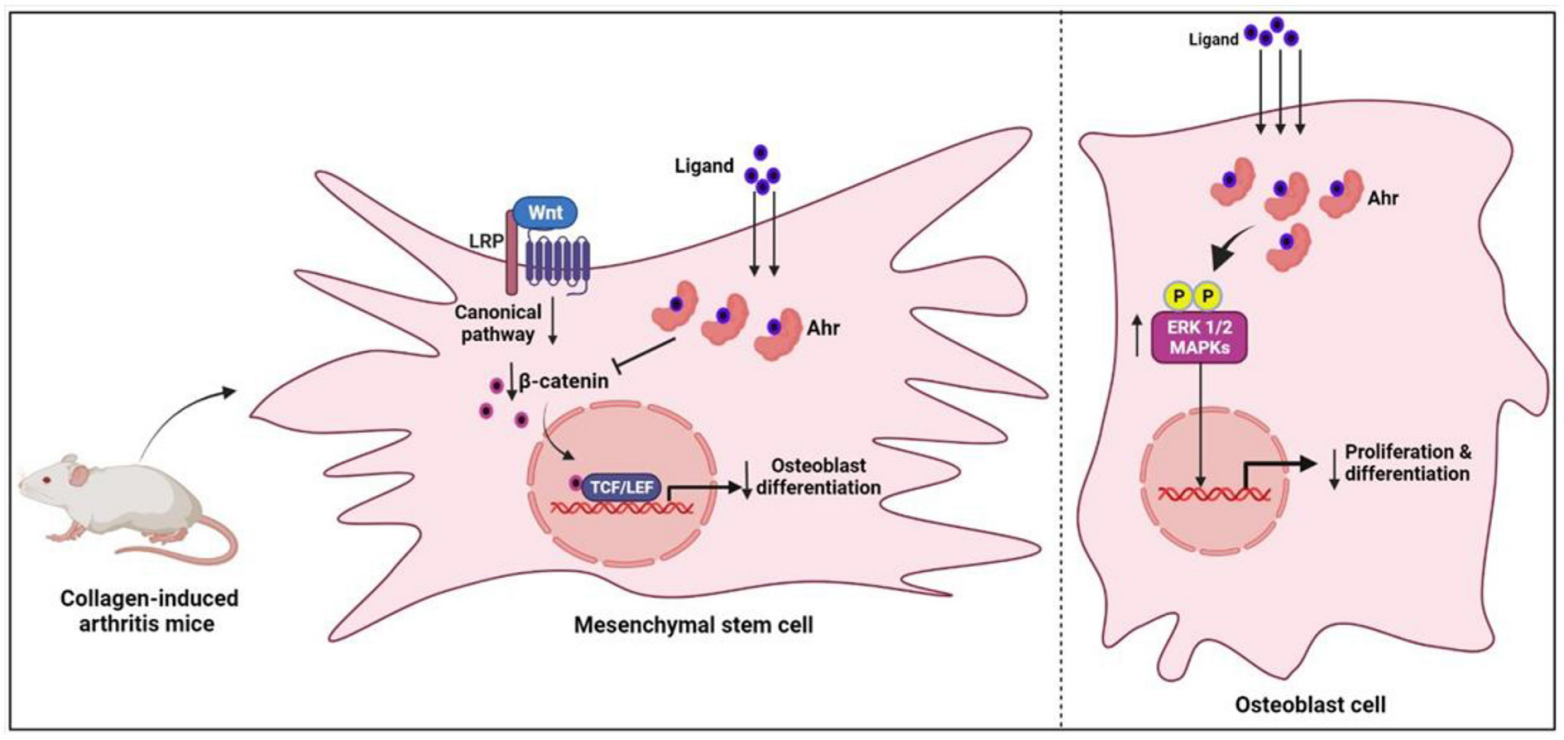
The mechanism of osteogenesis inhibition by Ahr-associated mesenchymal stem cell *via* the Wnt/-catenin pathway. Ahr, aryl hydrocarbon receptor; ERK1/2, extracellular signal_regulated protein kinase; MAPK, mitogen-activated protein kinase.

Aryl hydrocarbon receptor (Ahr) is a ligand-activated transcription factor that belongs to the Per-Arnt-Sim protein superfamily. It is a nuclear receptor and usually stays inactive in the cytosol due to its binding with cochaperones. When it binds to a specific ligand, its structure transforms and it is translocated to the nucleus, where it forms heterodimerization with aromatic hydrocarbon receptor nuclear transfer protein (ARNT). Subsequently, the Ahr/ARNT complex regulates biological functions, including toxicity, biological evolution, bone remodeling, and immune response by promoting gene transcription of various prototypic genes (70). Increasing evidence confirms its close association with the pathogenesis of immune diseases, including RA. Ahr activation significantly contributes to the development of RA (71). In experimental studies, Ahr activation with its agonists contributed to RA disease progression, bone damage and osteoclast differentiation (72). Ahr expression was found to be 2-fold higher in RA patients when compared to controls. Furthermore, a positive association between cigarette smoking and Ahr activation in RA patients has been demonstrated (73) (Figure 5).

Maintaining homeostasis in bone remodeling requires a balance between the relative activity of bone-forming osteoblasts and bone-resorbing osteoclasts. The modulation of AhR signaling *via* the NF-B, Wnt, and MAP kinase pathways has been linked to changes in bone remodeling (74–78). The activated Ahr may affect bone remodeling by interfering with the functional differentiation of osteoblasts and osteoclasts (79). The influence of Ahr signaling on osteoblast function is well–established in recent studies. The bone erosion in arthritic mice was found to be positively associated with high Ahr expression, and these highly expressed Ahr levels were relatively localized to the osteoblast cells (80). Additionally, in *in vitro* conditions, the activated Ahr inhibited the proliferation and differentiation of osteoblast cells by upregulating the ERK/MAPK signaling pathway (80). In further investigations, it was concluded that the activated Ahr in mesenchymal stem cells, derived from collagen-induced arthritis mice, inhibited osteogenesis by downregulating β-catenin (81). On the other hand, the influence of Ahr signaling on osteoclastogenesis is not clearly understood. In *in vivo* and *in vitro* studies of rodents, there were inconsistent results regarding the effects of Ahr on osteoclast differentiation (82). However, findings from a recent study on human osteoclast cells revealed that Ahr activation through kynurenine inhibited osteoclast differentiation by downregulating NFATc1 protein expression (83). The same study concluded that Ahr activation could be a potential target to treat the bone loss in RA. In support of this, natural products derived Ahr ligands/agonists have been shown to prevent bone erosions in RA (84, 85). Overall, the influence of Ahr signaling on RA-associated bone erosion is still controversial as it inhibits the osteoblast cells and may either inhibit or promote osteoclast differentiation.

## Disease-modifying anti-rheumatic drugs (DMARDs) and bone loss

Disease-modifying anti-rheumatic drugs (DMARDs) are the mainstay of treatment in RA. There are conventional (methotrexate, leflunomide, sulfasalazine), biologic (adalimumab, etanercept, golimumab, tocilizumab etc) and targeted synthetic DMARDs (tofacitinib, baricitinib, upadacitib). Apart from suppression of inflammation, there is ample evidence that all of these classes of drugs prevent articular erosions and radiographic progression of joint damage. Chen et al. reported that after 3 years of biologic or targeted synthetic DMARD therapy, BMD remained stable at the femoral neck, hip and lumbar vertebra (86). These findings were echoed by other studies (87, 88).

## Conclusions

There is compelling evidence that the interplay of various pathways and different mechanisms contribute to bone loss and secondary osteoporosis in RA. Osteoclastogenesis may occur *via* RANKL-dependent and RANKL-independent processes. TNFα, IL-1, IL-6 anti-CCP which are the key orchestrators of inflammation tend to affect bone remodeling adversely.

## Author contributions

RS, RU, K-YC, and SD contributed to the literature search and manuscript composition. SS contributed to creation of the figures and manuscript composition. All authors contributed to the article and approved the submitted version.

## Funding

This research was funded by a grant by the Ministry of Higher Education of Malaysia (FRGS/1/2021/SKK08/UKM/02/1) and the National University of Malaysia.

## Conflict of interest

The authors declare that the research was conducted in the absence of any commercial or financial relationships that could be construed as a potential conflict of interest.

## Publisher's note

All claims expressed in this article are solely those of the authors and do not necessarily represent those of their affiliated organizations, or those of the publisher, the editors and the reviewers. Any product that may be evaluated in this article, or claim that may be made by its manufacturer, is not guaranteed or endorsed by the publisher.

## References

[1] Eriksen EF. Cellular mechanisms of bone remodeling. Rev Endocr Metab Disord. (2010) 11:219–27. 10.1007/s11154-010-9153-1 21188536 PMC3028072

[2] Nistala H Lee-Arteaga S Siciliano G Smaldone S Ramirez F. Extracellular regulation of transforming growth factor beta and bone morphogenetic protein signaling in bone. Ann N Y Acad Sci. (2010) 1192:253–6. 10.1111/j.1749-6632.2009.05350.x 20392244

[3] Wu X Shi W Cao X. Multiplicity of BMP signaling in skeletal development. Ann N Y Acad Sci. (2007) 1116:29–49. 10.1196/annals.1402.053 18083919

[4] Pederson L Ruan M Westendorf JJ Khosla S Oursler MJ. Regulation of bone formation by osteoclasts involves Wnt/BMP signaling and the chemokine sphingosine-1-phosphate. Proc Natl Acad Sci U S A. (2008) 105:20764–9. 10.1073/pnas.0805133106 19075223 PMC2603259

[5] Negishi-Koga T Shinohara M Komatsu N Bito H Kodama T Friedel RH. Suppression of bone formation by osteoclastic expression of semaphorin 4D. Nat Med. (2011) 17:1473–80. 10.1038/nm.2489 22019888

[6] Sargin G Kose R Senturk T. Relationship between bone mineral density and anti-citrullinated protein antibody and rheumatoid factor in patients with rheumatoid arthritis. Eur J Rheumatol. (2019) 6:29–33. 10.5152/eurjrheum.2018.18099 30973322 PMC6459337

[7] Araki Y Mimura T. Matrix metalloproteinase gene activation resulting from disordred epigenetic mechanisms in rheumatoid arthritis. Int J Mol Sci. (2017) 18:905. 10.3390/ijms18050905 28441353 PMC5454818

[8] Green MJ Gough AK Devlin J Smith J Astin P Taylor D. Serum MMP-3 and MMP-1 and progression of joint damage in early rheumatoid arthritis. Rheumatology (Oxford). (2003) 42:83–8. 10.1093/rheumatology/keg037 12509618

[9] Di Spigna G Rossi FW Mormile I Ladogana P Buonavolonta L Covelli B. Serum Metalloprotease 3 (MMP-3) biomarker of therapeutic efficacy during treatment of rheumatoid arthritis. J Biol Regul Homeost Agents. (2021) 35:1041–5. 10.23812/21-86-L 34121372

[10] Sun Y Huang Y Chen T Li X Chen J Wang Z. Effect of downregulation of serum MMP-3 levels by traditional Chinese medicine ingredients combined with methotrexate on the progression of bone injury in patients with rheumatoid arthritis: A protocol for a systematic review and meta-analysis. Medicine (Baltimore). (2020) 99:e22841. 10.1097/MD.0000000000022841 33120813 PMC7581142

[11] Llorente I García-Castañeda N Valero C González-Álvaro I Castañeda S. Osteoporosis in rheumatoid arthritis: dangerous liaisons. Front Med (Lausanne). (2020) 7:601618. 10.3389/fmed.2020.601618 33330566 PMC7719815

[12] Goldring SR. The effects of inflammatory arthritis on bone remodeling. Arthritis Res Ther. (2005) 7:S12. 10.1186/ar1518

[13] Rivollier A Mazzorana M Tebib J Piperno M Aitsiselmi T Rabourdin-Combe C. Immature dendritic cell transdifferentiation into osteoclasts: a novel pathway sustained by the rheumatoid arthritis microenvironment. Blood. (2004) 104:4029–37. 10.1182/blood-2004-01-0041 15308576

[14] O'Brien CA. Control of RANKL gene expression. Bone. (2010) 46:911–9. 10.1016/j.bone.2009.08.050 19716455 PMC2842447

[15] Li Y Toraldo G Li A Yang X Zhang H Qian WP. B cells and T cells are critical for the preservation of bone homeostasis and attainment of peak bone mass in vivo. Blood. (2007) 109:3839–48. 10.1182/blood-2006-07-037994 17202317 PMC1874582

[16] Kong YY Yoshida H Sarosi I Tan HL Timms E Capparelli C. OPGL is a key regulator of osteoclastogenesis, lymphocyte development and lymph-node organogenesis. Nature. (1999) 397:315–23. 10.1038/16852 9950424

[17] Braun T Zwerina J. Positive regulators of osteoclastogenesis and bone resorption in rheumatoid arthritis. Arthritis Res Ther. (2011) 13:235. 10.1186/ar3380 21861862 PMC3239343

[18] Ono T Hayashi M Sasaki F Nakashima T. RANKL biology: bone metabolism, the immune system, and beyond. Inflamm Regen. (2020) 40:2. 10.1186/s41232-019-0111-3 32047573 PMC7006158

[19] Xiong J Piemontese M Onal M Campbell J Goellner JJ Dusevich V. Osteocytes, not osteoblasts or lining cells, are the main source of the RANKL required for osteoclast formation in remodeling bone. PLoS ONE. (2015) 10:e0138189. 10.1371/journal.pone.0138189 26393791 PMC4578942

[20] Kim KW Cho ML Oh HJ Kim HR Kang CM Heo YM. TLR-3 enhances osteoclastogenesis through upregulation of RANKL expression from fibroblast-like synoviocytes in patients with rheumatoid arthritis. Immunol Lett. (2009) 124:9–17. 10.1016/j.imlet.2009.02.006 19446344

[21] Komatsu N Win S Yan M Huynh NC Sawa S Tsukasaki M. Plasma cells promote osteoclastogenesis and periarticular bone loss in autoimmune arthritis. J Clin Invest. (2021) 131. 10.1172/JCI143060 33720039 PMC7954598

[22] Park JH Lee NK Lee SY. Current understanding of RANK signaling in osteoclast differentiation and maturation. Mol Cells. (2017) 40:706–13. 10.14348/molcells.2017.0225 29047262 PMC5682248

[23] Walsh MC Choi Y. Biology of the RANKL-RANK-OPG system in immunity, bone, and beyond. Front Immunol. (2014) 5:511. 10.3389/fimmu.2014.00511 25368616 PMC4202272

[24] Boyce BF Xing L. Biology of RANK, RANKL, and osteoprotegerin. Arthritis Res Ther. (2007) 9 (Suppl. 1):S1. 10.1186/ar2165 17634140 PMC1924516

[25] Saidenberg-Kermanac'h N Cohen-Solal M Bessis N De Vernejoul MC Boissier MC. Role for osteoprotegerin in rheumatoid inflammation. Joint Bone Spine. (2004) 71:9–13. 10.1016/S1297-319X(03)00131-3 14769514

[26] Hashizume M Hayakawa N Mihara M. IL-6 trans-signalling directly induces RANKL on fibroblast-like synovial cells and is involved in RANKL induction by TNF-alpha and IL-17. Rheumatology (Oxford). (2008) 47:1635–40. 10.1093/rheumatology/ken363 18786965

[27] Takeshita A Nishida K Yoshida A Nasu Y Nakahara R Kaneda D. RANKL expression in chondrocytes and its promotion by lymphotoxin-α in the course of cartilage destruction during rheumatoid arthritis. PLoS ONE. (2021) 16:e0254268. 10.1371/journal.pone.0254268 34234380 PMC8263262

[28] Pettit AR Walsh NC Manning C Goldring SR Gravallese EM. RANKL protein is expressed at the pannus-bone interface at sites of articular bone erosion in rheumatoid arthritis. Rheumatology (Oxford). (2006) 45:1068–76. 10.1093/rheumatology/kel045 16490750

[29] Danks L Komatsu N Guerrini MM Sawa S Armaka M Kollias G. RANKL expressed on synovial fibroblasts is primarily responsible for bone erosions during joint inflammation. Ann Rheum Dis. (2016) 75:1187–95. 10.1136/annrheumdis-2014-207137 26025971

[30] Perpétuo IP Caetano-Lopes J Rodrigues AM Campanilho-Marques R Ponte C Canhão H. Methotrexate and low-dose prednisolone downregulate osteoclast function by decreasing receptor activator of nuclear factor-κβ expression in monocytes from patients with early rheumatoid arthritis. RMD Open. (2017) 3:e000365. 10.1136/rmdopen-2016-000365 28955481 PMC5604603

[31] Boman A Kokkonen H Ärlestig L Berglin E Rantapää-Dahlqvist S. Receptor activator of nuclear factor kappa-B ligand (RANKL) but not sclerostin or gene polymorphisms is related to joint destruction in early rheumatoid arthritis. Clin Rheumatol. (2017) 36:1005–12. 10.1007/s10067-017-3570-4 28190118 PMC5400786

[32] Yang H Liu W Zhou X Rui H Zhang H Liu R. The association between RANK, RANKL and OPG gene polymorphisms and the risk of rheumatoid arthritis: a case-controlled study and meta-analysis. Biosci Rep. (2019) 39:BSR20182356. 10.1042/BSR20182356 31209146 PMC6597846

[33] Wielińska J Kolossa K Swierkot J Dratwa M Iwaszko M Bugaj B. Polymorphisms within the RANK and RANKL encoding genes in patients with rheumatoid arthritis: association with disease progression and effectiveness of the biological treatment. Arch Immunol Ther Exp (Warsz). (2020) 68:24. 10.1007/s00005-020-00590-6 32815001 PMC7438366

[34] Ruyssen-Witrand A Degboé Y Cantagrel A Nigon D Lukas C Scaramuzzino S. Association between RANK, RANKL and OPG polymorphisms with ACPA and erosions in rheumatoid arthritis: results from a meta-analysis involving three French cohorts. RMD Open. (2016) 2:e000226-e. 10.1136/rmdopen-2015-000226 27651922 PMC5020667

[35] So H Cheng IT Lau SL Chow E Lam T Hung VW. Effects of RANKL inhibition on promoting healing of bone erosion in rheumatoid arthritis using HR-pQCT: a 2-year, randomised, double-blind, placebo-controlled trial. Ann Rheum Dis. (2021) 80:981–8. 10.1136/annrheumdis-2021-219846 33811034

[36] Takeuchi T Tanaka Y Soen S Yamanaka H Yoneda T Tanaka S. Effects of the anti-RANKL antibody denosumab on joint structural damage in patients with rheumatoid arthritis treated with conventional synthetic disease-modifying antirheumatic drugs (DESIRABLE study): a randomised, double-blind, placebo-controlled phase 3 trial. Ann Rheum Dis. (2019) 78:899–907. 10.1136/annrheumdis-2018-214827 31036625 PMC6585575

[37] Saag KG McDermott MT Adachi J Lems W Lane NE Geusens P. The effect of discontinuing denosumab in patients with rheumatoid arthritis treated with glucocorticoids. Arthritis Rheumatol. (2022) 74:604–11. 10.1002/art.41981 34535967 PMC9305881

[38] Romas E Sims NA Hards DK Lindsay M Quinn JW Ryan PF. Osteoprotegerin reduces osteoclast numbers and prevents bone erosion in collagen-induced arthritis. Am J Pathol. (2002) 161:1419–27. 10.1016/S0002-9440(10)64417-3 12368214 PMC1867274

[39] van Tuyl LH Voskuyl AE Boers M Geusens P Landewé RB Dijkmans BA. Baseline RANKL:OPG ratio and markers of bone and cartilage degradation predict annual radiological progression over 11 years in rheumatoid arthritis. Ann Rheum Dis. (2010) 69:1623–8. 10.1136/ard.2009.121764 20525836

[40] Xu S Wang Y Lu J Xu J. Osteoprotegerin and RANKL in the pathogenesis of rheumatoid arthritis-induced osteoporosis. Rheumatol Int. (2012) 32:3397–403. 10.1007/s00296-011-2175-5 22057136

[41] Zhao B. TNF and bone remodeling. Curr Osteoporos Rep. (2017) 15:126–34. 10.1007/s11914-017-0358-z 28477234 PMC6408950

[42] Zerbini CAF Clark P Mendez-Sanchez L Pereira RMR Messina OD Uña CR. Biologic therapies and bone loss in rheumatoid arthritis. Osteoporos Int. (2017) 28:429–46. 10.1007/s00198-016-3769-2 27796445

[43] Jura-Poltorak A Szeremeta A Olczyk K Zon-Giebel A Komosinska-Vassev K. Bone metabolism and RANKL/OPG ratio in rheumatoid arthritis women treated with TNF-alpha inhibitors. J Clin Med. (2021) 10:2905. 10.3390/jcm10132905 34209821 PMC8267676

[44] Perpetuo IP Caetano-Lopes J Rodrigues AM Campanilho-Marques R Ponte C Canhao H. Effect of tumor necrosis factor inhibitor therapy on osteoclasts precursors in rheumatoid arthritis. Biomed Res Int. (2017) 2017:2690402. 10.1155/2017/2690402 28286757 PMC5327780

[45] Yoshitake F Itoh S Narita H Ishihara K Ebisu S. Interleukin-6 directly inhibits osteoclast differentiation by suppressing receptor activator of NF-kappaB signaling pathways. J Biol Chem. (2008) 283:11535–40. 10.1074/jbc.M607999200 18296709

[46] Bettelli E Carrier Y Gao W Korn T Strom TB Oukka M. Reciprocal developmental pathways for the generation of pathogenic effector TH17 and regulatory T cells. Nature. (2006) 441:235–8. 10.1038/nature04753 16648838

[47] Moser T Akgun K Proschmann U Sellner J Ziemssen T. The role of TH17 cells in multiple sclerosis: therapeutic implications. Autoimmun Rev. (2020) 19:102647. 10.1016/j.autrev.2020.102647 32801039

[48] Sato K Suematsu A Okamoto K Yamaguchi A Morishita Y Kadono Y. Th17 functions as an osteoclastogenic helper T cell subset that links T cell activation and bone destruction. J Exp Med. (2006) 203:2673–82. 10.1084/jem.20061775 17088434 PMC2118166

[49] Matsuura Y Kikuta J Kishi Y Hasegawa T Okuzaki D Hirano T. In vivo visualisation of different modes of action of biological DMARDs inhibiting osteoclastic bone resorption. Ann Rheum Dis. (2018) 77:1219–25. 10.1136/annrheumdis-2017-212880 29705743

[50] O'Brien W Fissel BM Maeda Y Yan J Ge X Gravallese EM. RANK-independent osteoclast formation and bone erosion in inflammatory arthritis. Arthritis Rheumatol. (2016) 68:2889–900. 10.1002/art.39837 27563728 PMC5125876

[51] Garlanda C Dinarello CA Mantovani A. The interleukin-1 family: back to the future. Immunity. (2013) 39:1003–18. 10.1016/j.immuni.2013.11.010 24332029 PMC3933951

[52] Dinarello CA. Overview of the interleukin-1 family of ligands and receptors. Semin Immunol. (2013) 25:389–93. 10.1016/j.smim.2013.10.001 24275600

[53] Nakamura I Jimi E. Regulation of osteoclast differentiation and function by interleukin-1. Vitam Horm. (2006) 74:357–70. 10.1016/S0083-6729(06)74015-8 17027523

[54] Ruscitti P Cipriani P Carubbi F Liakouli V Zazzeroni F Di Benedetto P. The role of IL-1β in the bone loss during rheumatic diseases. Mediators Inflamm. (2015) 2015:782382. 10.1155/2015/782382 25954061 PMC4410538

[55] Lorenzo J Horowitz M Choi Y. Osteoimmunology: interactions of the bone and immune system. Endocr Rev. (2008) 29:403–40. 10.1210/er.2007-0038 18451259 PMC2528852

[56] Lacativa PG Farias ML. Osteoporosis and inflammation. Arq Bras Endocrinol Metabol. (2010) 54:123–32. 10.1590/S0004-27302010000200007 20485900

[57] Redlich K Smolen JS. Inflammatory bone loss: pathogenesis and therapeutic intervention. Nat Rev Drug Discov. (2012) 11:234–50. 10.1038/nrd3669 22378270

[58] Chen HY Chen WC Wu MC Tsai FJ Lin CC. Interleukin-1beta and interleukin-1 receptor antagonist gene polymorphism in postmenopausal women: correlation to bone mineral density and susceptibility to osteoporosis. Maturitas. (2003) 44:49–54. 10.1016/S0378-5122(02)00313-4 12568735

[59] Ganesan R Rasool M. Interleukin 17 regulates SHP-2 and IL-17RA/STAT-3 dependent Cyr61, IL-23 and GM-CSF expression and RANKL mediated osteoclastogenesis by fibroblast-like synoviocytes in rheumatoid arthritis. Mol Immunol. (2017) 91:134–44. 10.1016/j.molimm.2017.09.003 28898718

[60] Kar S Gupta R Malhotra R Sharma V Farooque K Kumar V. Interleukin-9 facilitates osteoclastogenesis in rheumatoid arthritis. Int J Mol Sci. (2021) 22:10397. 10.3390/ijms221910397 34638736 PMC8508938

[61] Min HK Won JY Kim BM Lee KA Lee SJ Lee SH. Interleukin (IL)-25 suppresses IL-22-induced osteoclastogenesis in rheumatoid arthritis via STAT3 and p38 MAPK/IkappaBalpha pathway. Arthritis Res Ther. (2020) 22:222. 10.1186/s13075-020-02315-8 32972460 PMC7517649

[62] Choi S Lee KH. Clinical management of seronegative and seropositive rheumatoid arthritis: a comparative study. PLoS ONE. (2018) 13:e0195550. 10.1371/journal.pone.0195550 29624625 PMC5889180

[63] Kurowska W Slowinska I Krogulec Z Syrowka P Maslinski W. Antibodies to citrullinated proteins (ACPA) associate with markers of osteoclast activation and bone destruction in the bone marrow of patients with rheumatoid arthritis. J Clin Med. (2021) 10:1778. 10.3390/jcm10081778 33921836 PMC8073027

[64] Bugatti S Bogliolo L Vitolo B Manzo A Montecucco C Caporali R. Anti-citrullinated protein antibodies and high levels of rheumatoid factor are associated with systemic bone loss in patients with early untreated rheumatoid arthritis. Arthritis Res Ther. (2016) 18:226. 10.1186/s13075-016-1116-9 27716332 PMC5052789

[65] Llorente I Merino L Ortiz AM Escolano E González-Ortega S García-Vicuña R. Anti-citrullinated protein antibodies are associated with decreased bone mineral density: baseline data from a register of early arthritis patients. Rheumatol Int. (2017) 37:799–806. 10.1007/s00296-017-3674-9 28243799 PMC5397447

[66] Lv Y Wang G Xu W Tao P Lv X. Wang Y. Tartrate-resistant acid phosphatase 5b is a marker of osteoclast number and volume in RAW 2647 cells treated with receptor-activated nuclear κB ligand. Exp Ther Med. (2015) 9:143–6. 10.3892/etm.2014.2071 25452790 PMC4247282

[67] Goto T Yamaza T Tanaka T. Cathepsins in the osteoclast. J Electron Microsc (Tokyo). (2003) 52:551–8. 10.1093/jmicro/52.6.551 14756243

[68] Krishnamurthy A Joshua V Haj Hensvold A Jin T Sun M Vivar N. Identification of a novel chemokine-dependent molecular mechanism underlying rheumatoid arthritis-associated autoantibody-mediated bone loss. Ann Rheum Dis. (2016) 75:721–9. 10.1136/annrheumdis-2015-208093 26612338 PMC4819614

[69] Regueiro C Ortiz AM Boveda MD Castañeda S Gonzalez-Alvaro I Gonzalez A. Association of high titers of anti-carbamylated protein antibodies with decreased bone mineral density in early arthritis patients. PLoS ONE. (2018) 13:e0202583. 10.1371/journal.pone.0202583 30118518 PMC6097678

[70] Yu H Jiang L Wan B Zhang W Yao L Che T. The role of aryl hydrocarbon receptor in bone remodeling. Prog Biophys Mol Biol. (2018) 134:44–9. 10.1016/j.pbiomolbio.2017.12.005 29277341

[71] Nguyen NT Nakahama T Kishimoto T. Aryl hydrocarbon receptor and experimental autoimmune arthritis. Semin Immunopathol. (2013) 35:637–44. 10.1007/s00281-013-0392-6 23982178

[72] Fu J Nogueira SV Drongelen VV Coit P Ling S Rosloniec EF. Shared epitope-aryl hydrocarbon receptor crosstalk underlies the mechanism of gene-environment interaction in autoimmune arthritis. Proc Natl Acad Sci U S A. (2018) 115:4755–60. 10.1073/pnas.1722124115 29666259 PMC5939100

[73] Kazantseva MG Highton J Stamp LK Hessian PA. Dendritic cells provide a potential link between smoking and inflammation in rheumatoid arthritis. Arthritis Res Ther. (2012) 14:R208. 10.1186/ar4046 23036591 PMC3580520

[74] Øvrevik J Låg M Lecureur V Gilot D Lagadic-Gossmann D Refsnes M. AhR and Arnt differentially regulate NF-κB signaling and chemokine responses in human bronchial epithelial cells. Cell Commun Signal. (2014) 12:48. 10.1186/s12964-014-0048-8 25201625 PMC4222560

[75] Schneider AJ Branam AM Peterson RE. Intersection of AHR and Wnt signaling in development, health, and disease. Int J Mol Sci. (2014) 15:17852–85. 10.3390/ijms151017852 25286307 PMC4227194

[76] Wang Q Kurita H Carreira V Ko CI Fan Y Zhang X. Ah receptor activation by dioxin disrupts activin, BMP, and WNT signals during the early differentiation of mouse embryonic stem cells and inhibits cardiomyocyte functions. Toxicol Sci. (2016) 149:346–57. 10.1093/toxsci/kfv246 26572662 PMC4900218

[77] Wincent E Stegeman JJ Jönsson ME. Combination effects of AHR agonists and Wnt/β-catenin modulators in zebrafish embryos: Implications for physiological and toxicological AHR functions. Toxicol Appl Pharmacol. (2015) 284:163–79. 10.1016/j.taap.2015.02.014 25711857 PMC4747639

[78] Occhi G Barollo S Regazzo D Bertazza L Galuppini F Guzzardo V. A constitutive active MAPK/ERK pathway due to BRAFV600E positively regulates AHR pathway in PTC. Oncotarget. (2015) 6:32104–14. 10.18632/oncotarget.5194 26392334 PMC4741662

[79] Korkalainen M Kallio E Olkku A Nelo K Ilvesaro J Tuukkanen J. Dioxins interfere with differentiation of osteoblasts and osteoclasts. Bone. (2009) 44:1134–42. 10.1016/j.bone.2009.02.019 19264158

[80] Yu H Du Y Zhang X Sun Y Li S Dou Y. The aryl hydrocarbon receptor suppresses osteoblast proliferation and differentiation through the activation of the ERK signaling pathway. Toxicol Appl Pharmacol. (2014) 280:502–10. 10.1016/j.taap.2014.08.025 25194622

[81] Tong Y Niu M Du Y Mei W Cao W Dou Y. Aryl hydrocarbon receptor suppresses the osteogenesis of mesenchymal stem cells in collagen-induced arthritic mice through the inhibition of β-catenin. Exp Cell Res. (2017) 350:349–57. 10.1016/j.yexcr.2016.12.009 28007558

[82] Park R Madhavaram S Ji JD. The role of aryl-hydrocarbon receptor (AhR) in osteoclast differentiation and function. Cells. (2020) 9:2294. 10.3390/cells9102294 33066667 PMC7602422

[83] Kim SY Oh Y Jo S Ji JD Kim TH. Inhibition of human osteoclast differentiation by kynurenine through the aryl-hydrocarbon receptor pathway. Cells. (2021) 10:3498. 10.3390/cells10123498 34944003 PMC8700497

[84] Tong B Yuan X Dou Y Wu X Wang Y Xia Y. Sinomenine induces the generation of intestinal Treg cells and attenuates arthritis via activation of aryl hydrocarbon receptor. Lab Invest. (2016) 96:1076–86. 10.1038/labinvest.2016.86 27617398

[85] Tong B Yuan X Dou Y Wu X Chou G Wang Z. Norisoboldine, an isoquinoline alkaloid, acts as an aryl hydrocarbon receptor ligand to induce intestinal Treg cells and thereby attenuate arthritis. Int J Biochem Cell Biol. (2016) 75:63–73. 10.1016/j.biocel.2016.03.014 27032495

[86] Chen JF Hsu CY Yu SF Ko CH Chiu WC Lai HM. The impact of long-term biologics/target therapy on bone mineral density in rheumatoid arthritis: a propensity score-matched analysis. Rheumatology (Oxford). (2020) 59:2471–80. 10.1093/rheumatology/kez655 31984422 PMC7449814

[87] Chen MH Yu SF Chen JF Chen WS Liou TL Chou CT. Different effects of biologics on systemic bone loss protection in rheumatoid arthritis: an interim analysis of a three-year longitudinal cohort study. Front Immunol. (2021) 12:783030. 10.3389/fimmu.2021.783030 34987510 PMC8720866

[88] Chen YM Chen HH Huang WN Liao TL Chen JP Chao WC. Tocilizumab potentially prevents bone loss in patients with anticitrullinated protein antibody-positive rheumatoid arthritis. PLoS ONE. (2017) 12:e0188454. 10.1371/journal.pone.0188454 29155868 PMC5695761

[89] Hu Q Zhong X Tian H Liao P. The efficacy of denosumab in patients with rheumatoid arthritis: a systematic review and pooled analysis of randomized or matched data. Front Immunol. (2021) 12:799575. 10.3389/fimmu.2021.799575 35069583 PMC8766643

[90] Polzer K Joosten L Gasser J Distler JH Ruiz G Baum W. Interleukin-1 is essential for systemic inflammatory bone loss. Ann Rheum Dis. (2010) 69:284–90. 10.1136/ard.2008.104786 19196726

[91] Gulyás K Horváth Á Végh E Pusztai A Szentpétery Á Pethö Z. Effects of 1-year anti-TNF-α therapies on bone mineral density and bone biomarkers in rheumatoid arthritis and ankylosing spondylitis. Clin Rheumatol. (2020) 39:167–75. 10.1007/s10067-019-04771-3 31522318

[92] Zwerina J Hayer S Tohidast-Akrad M Bergmeister H Redlich K Feige U. Single and combined inhibition of tumor necrosis factor, interleukin-1, and RANKL pathways in tumor necrosis factor-induced arthritis: effects on synovial inflammation, bone erosion, and cartilage destruction. Arthritis Rheum. (2004) 50:277–90. 10.1002/art.11487 14730626

[93] Binder NB Puchner A Niederreiter B Hayer S Leiss H Blüml S. Tumor necrosis factor-inhibiting therapy preferentially targets bone destruction but not synovial inflammation in a tumor necrosis factor-driven model of rheumatoid arthritis. Arthritis Rheum. (2013) 65:608–17. 10.1002/art.37797 23280418

[94] Axmann R Böhm C Krönke G Zwerina J Smolen J Schett G. Inhibition of interleukin-6 receptor directly blocks osteoclast formation *in vitro* and *in vivo*. Arthritis Rheum. (2009) 60:2747–56. 10.1002/art.24781 19714627

[95] Lange U Teichmann J Müller-Ladner U Strunk J. Increase in bone mineral densit y of patients with rheumatoid arthritis treated with anti-TNF-alpha antibody: a prospective open-label pilot study. Rheumatology (Oxford). (2005) 44:1546–8. 10.1093/rheumatology/kei082 16263785

[96] Cheng TT Yu SF Su FM Chen YC Su BY Chiu WC. Anti-CCP-positive patients with RA have a higher 10-year probability of fracture evaluated by FRAX®: a registry study of RA with osteoporosis/fracture. Arthritis Res Ther. (2018) 20:16. 10.1186/s13075-018-1515-1 29382355 PMC5791167

[97] Hafström I Ajeganova S Andersson ML Bala SV Bergman S Bremander A. A Swedish register-based, long-term inception cohort study of patients with rheumatoid arthritis - results of clinical relevance. Open Access Rheumatol. (2019) 11:207–17. 10.2147/OARRR.S218448 31565006 PMC6744369

[98] Wysham KD Shoback DM Imboden JB Katz PP. Association of high anti-cyclic citrullinated peptide seropositivity and lean mass index with low bone mineral density in rheumatoid arthritis. Arthritis Care Res (Hoboken). (2018) 70:961–9. 10.1002/acr.23440 29106028 PMC5936473

[99] Amkreutz J de Moel EC Theander L Willim M Heimans L Nilsson J. Association between bone mineral density and autoantibodies in patients with rheumatoid arthritis. Arthritis Rheumatol. (2021) 73:921–30. 10.1002/art.41623 33314699 PMC8251600

[100] Ahmad HA Alemao E Guo Z Iannaccone CK Frits ML Weinblatt M. Association of low bone mineral density with anti-citrullinated protein antibody positivity and disease activity in established rheumatoid arthritis: findings from a US observational cohort. Adv Ther. (2018) 35:232–42. 10.1007/s12325-017-0657-x 29368271 PMC5818577

